# Identification of markers of prostate cancer progression using candidate gene expression

**DOI:** 10.1038/bjc.2011.490

**Published:** 2011-11-10

**Authors:** S E T Larkin, S Holmes, I A Cree, T Walker, V Basketter, B Bickers, S Harris, S D Garbis, P A Townsend, C Aukim-Hastie

**Affiliations:** 1 School of Pharmacy and Biomedical Sciences, University of Portsmouth, St Michaels Building, White Swan Road, Portsmouth, PO1 2DT, UK; 2 Department of Urology, Queen Alexandra Hospital, Portsmouth, PO6 3LY, UK; 3 Translational Oncology Research Centre, Queen Alexandra Hospital, Portsmouth, PO6 3LY, UK; 4 Public Health Sciences and Medical Statistics, University of Southampton, Southampton, UK; 5 Biomedical Research Foundation, Academy of Athens, Athens, Greece; 6 Cancer Sciences Unit, Faculty of Medicine, University of Southampton, Southampton, UK

**Keywords:** Taqman, qPCR, prostate cancer, *ANPEP*, *ABL1*, *PSCA*, *TRIP13*

## Abstract

**Background:**

Metastatic prostate cancer (PCa) has no curative treatment options. Some forms of PCa are indolent and slow growing, while others metastasise quickly and may prove fatal within a very short time. The basis of this variable prognosis is poorly understood, despite considerable research. The aim of this study was to identify markers associated with the progression of PCa.

**Methods:**

Artificial neuronal network analysis combined with data from literature and previous work produced a panel of putative PCa progression markers, which were used in a transcriptomic analysis of 29 radical prostatectomy samples and correlated with clinical outcome.

**Results:**

Statistical analysis yielded seven putative markers of PCa progression, *ANPEP, ABL1, PSCA, EFNA1, HSPB1, INMT* and *TRIP13.* Two data transformation methods were utilised with only markers that were significant in both selected for further analysis. *ANPEP* and *EFNA1* were significantly correlated with Gleason score. Models of progression co-utilising markers *ANPEP* and *ABL1* or *ANPEP* and *PSCA* had the ability to correctly predict indolent or aggressive disease, based on Gleason score, in 89.7% and 86.2% of cases, respectively. Another model of *TRIP13* expression in combination with preoperative PSA level and Gleason score was able to correctly predict recurrence in 85.7% of cases.

**Conclusion:**

This proof of principle study demonstrates a novel association of carcinogenic and tumourigenic gene expression with PCa stage and prognosis.

Prostate cancer (PCa) is the most common male cancer in the United Kingdom and accounts for 12% of male cancer deaths, making it the second most common cause of male cancer death (CRUK, 2010). At present, the use of markers such as PSA to indicate the presence of prostatic disease are severely flawed and lead to unnecessary biopsy procedures, putting patients at risk of infection and haemorrhage. In addition, many patients with high PSA levels are PCa negative on investigation of the biopsy and, conversely, PCa can occur without a rise in PSA and tends to be a more aggressive form of the disease. Once a diagnosis of PCa is confirmed, staging from a biopsy specimen is extremely problematic because of the multifocal nature of the disease. Inappropriate staging of the tumour may lead to radical procedures such as prostate excision, which can lead to a risk of: incontinence (35%), impotence (58%), infection, thrombosis or haemorrhage (Wilt *et al*, 2008). Approximately 20% of radical prostatectomy patients will recur indicating that a conservative treatment strategy might have been more appropriate if one could identify those patients at low risk of recurrence (Bill-Axelson *et al*, 2008). Equally, metastatic PCa has a variable prognosis with some patients suffering from a more indolent form of the disease with no significant impact on their quality of life or lifespan for at least 15 years. This has led to the use of monitoring strategies where patients’ PSA levels are monitored and biopsies taken if required. If there is evidence of PCa growth, radical prostatectomy or other treatments may be suggested (NICE, 2008). However, some patients suffer from a more aggressive form of PCa, with a median time to clinically apparent metastasis of 2 years (Siddiqui *et al*, 2004). These patients quickly develop local effects of urinary incontinence and pelvic pain and often present with late stage prostate disease, with local spread and occult metastasis, a stage at which there are no curative treatment options. Currently, there is no definitive method to differentiate indolent from aggressive disease. The use of watchful waiting strategies are more acceptable to patients but in some may lead to the unchecked growth and spread of a prostate tumour, especially of those more aggressive tumours, which do not express PSA.

Quantitative real-time PCR (qPCR) has become a useful tool for validation and reduction of data produced from microarray studies (VanGuilder *et al*, 2008). Many PCa biomarker studies employ microarrays to identify profiles of the disease and its progression (Chetcuti *et al*, 2001; Ding *et al*, 2006). However, these studies produce huge volumes of data that are difficult to analyse and resolve further. Chetcuti *et al* (2001) studied 588 genes in benign and malignant prostate tissue. They identified 87 genes that showed differential expression between the two tissue types. Ding *et al* (2006) studied 12 000 genes in normal and metastatic PCa cell lines. Both studies then utilised qPCR to further analyse the data produced by microarray analysis.

Standard qPCR is labour intensive, and therefore validation of >10–20 genes is rare. Taqman arrays present a medium-throughput method for qPCR, enabling the analysis of up to 383 genes per assay card (Abruzzo *et al*, 2005).

The aim of our current study was to identify putative PCa progression markers by using a panel of 91 genes and assessing expression levels in PCa tissue.

## Materials and methods

### Tissue specimens

Ethical approval was obtained and informed written consent was given by each patient included in this study. All PCa patients were from the Portsmouth region and were included if they had undergone radical prostatectomy between 2003 and 2006 and had no other cancers. Radical prostatectomy specimens were formalin fixed, paraffin embedded and stored within the Department of Pathology at Queen Alexandra Hospital following routine clinical analysis. A total of 29 tissue samples were successfully analysed: 18 Gleason score ⩾7 and 11 Gleason score <7, 20 without recurrence and 9 with recurrence. Specimen collection was based on the principles of scientific sampling as described by Garfield (2000) and other references therein. Although Gleason scoring is flawed as an indicator of progression, it still remains one of the best indicators of lymph node involvement, treatment failure and death from PCa (NICE, 2008) and as such is routinely recorded in clinic notes making this a well-documented endpoint to assess. Markers found to be differentially expressed between Gleason score, groupings may be indicative of a more aggressive phenotype so could be assessed in longer term survival studies.

### Identification of putative biomarkers

Taqman arrays were designed (genes included on the array are detailed in Supplementary Information, Supplementary Table 1) and ordered from Applied Biosystems (Carlsbad, CA, USA). The arrays were made up of a panel of putative PCa markers primarily derived from an analysis of published microarray data using artificial neuronal networks (ANNs) (Narayanan and Keedwell, 2006). Although the analysis by Narayanan and Keedwell (2006) focussed on diagnostic markers, it has been observed frequently that some diagnostic markers are altered during disease progression also. Therefore, this study was utilised to provide an unbiased panel for Taqman analysis. As ANNs are mathematical models that use complex statistical modelling to learn from and reduce large data sets, such as microarray data, the genes selected were more likely to contain markers of interest (Abbod *et al*, 2007). Narayanan and Keedwell (2006) utilised Affymetrix (Santa Clara, CA, USA) data from 102 samples (52 PCa cases and 50 normal) spanning 12 533 genes (Welsh *et al*, 2001) to assess the use of ‘perceptrons’ to identify the most important carcinogenesis genes within an extensive data set. A perceptron is an iterative algorithm that can inspect the effect of each gene on the output classification. Narayanan and Keedwell (2006) used two methods to reduce the genes to a core set of PCa-associated genes: a cross-validation method involving three rounds of iterative training with a cross-validation step after each iteration (102 samples divided into four groups); and an iteration only method, which just involved three rounds of iterative training, no cross-validation (102 samples divided into three groups). The methods employed were similar to those used previously to study melanoma and childhood medulloblastoma (Narayanan *et al*, 2004a, 2004b). The cross-validation method yielded 52 genes and the iteration only method yielded 44 genes, 21 of which were common to the cross-validation method. This study utilised the genes identified by the iteration only method to include in the panel of markers for qPCR analysis as this incorporated all of the samples for maximum knowledge discovery (Narayanan and Keedwell, 2006). Additional markers were included following a review of current literature to identify further putative PCa progression markers. Pubmed was used to search for PCa prognosis markers and progression markers. The most promising markers, based upon prostate biology and discussions with urologists and original authors, were included on the array (e.g., prostate stem cell antigen (PSCA)). In addition, markers identified from our previous work were also included, such as ANXA2 and CD9 (Hastie *et al*, 2005).

To provide information on inter- and intra-assay variation, reference genes included were included on each Taqman array: *18S*, *TBP* (TATA box-binding protein), *HPRT1* (hypoxanthine phosphoribosyltransferase 1), *PBGD* (porphobilinogen deaminase) and *SDHA* (succinate dehydrogenase complex, subunit A), which all showed little variation of expression in prostate and PCa tissues (Aerts *et al*, 2004; Ohl *et al*, 2005; Schmidt *et al*, 2006). *PBGD* in particular was chosen for normalisation as studies found it to show little variation between PCa tissue of different stage and grade.

### Identification and extraction of cancerous tissue

Tumour tissue was identified by a histopathologist and, to avoid inconsistency, the same histopathologist worked on this project throughout. Haematoxylin and eosin (HE) labelled slides were used to identify regions of the highest grade of PCa and these regions were marked. The HE slide was then used to guide sampling of the tissue of interest. Where possible, duplicate 0.6 mm punches were taken using a manual tissue microarray platform (Beecher Instruments, Mitogen, UK). A total of 41 samples were included at this stage.

### RNA extraction and two-step qPCR

RNA was extracted and reverse transcription (RT) performed as detailed by Glaysher *et al* (2009). Nucleic acid quantification was carried out using the Nanodrop 1000 spectrophotometer according to the instruction manual (Thermo Scientific, Waltham, MA, USA) and the concentrations and purity ratios were recorded. Newly prepared cDNA was subjected to an assessment of quality before large-scale qPCR analysis. *PBGD* (reference gene) was amplified by qPCR (using the iCycler, Bio-Rad, Hemel Hempstead, UK) in triplicate for each sample and also once for each RT-negative control. Additionally, several no template controls and a positive control in triplicate were included. These ‘sighting shot’ experiments were performed as detailed by Glaysher *et al* (2009). The experimental qPCR cycling parameters were; 1 cycle of 50 °C for 120 s and 95 °C for 600 s, then 50 cycles of 95 °C for 15 s and 60 °C for 60 s. A *C*_T_ (cycle threshold) of <40 was classed as showing good quality cDNA.

The cDNA was prepared and loaded, and the Taqman arrays sealed as described by Glaysher *et al* (2009) and qPCR performed using the following cycling parameters; heating to 50 °C for 120 s, further heating to 94.5 °C for 600 s and then 40 cycles of 97 °C for 30 s and 59.7 °C for 60 s. At the start and end of the trial, a standard curve plate of different dilutions of a pooled cDNA reference sample was performed to determine the efficiency of the assay. Additionally, the same pooled cDNA reference sample was used to perform a triplicate repeat at the start and end of the trial to assess intra- and inter-plate variability. Triplicate data could not be obtained for each sample because of insufficient sample quantity.

### Analysis of Taqman array data

Statistical analysis was performed using SPSS (version 12; IBM, Portsmouth, UK) on samples that showed a *C*_T_ of <35 for both *18S* and *PBGD*. Taqman array data were converted to normalised expression ratios using two methods; the first method was the Applied Biosystems recommended method (2^−ΔΔC_T_^) and the second was a method that allows for variation of amplification efficiency (Pfaffl method). Both methods were used to corroborate any findings by two independent analyses. First, the 2^−ΔΔC_T_^ method (Bio-Rad, 2006) was used followed by normalisation to PBGD and Ln (natural log) transformation. A Student's *t*-test was then performed to check for significant differences between the two groups. Second, the Pfaffl method was used (see equation in Supplementary Information, Supplementary Table 2), which takes into account the amplification efficiencies of the genes and normalises to a reference gene (*PBGD*) (Bio-Rad, 2006). This data conversion was also followed by Ln transformation and Student's *t*-test analysis. For both *t*-tests, genes showing differential expression between the indolent and aggressive groups (*P*<0.05) were considered for further analysis. Genes found to be significantly different were then assessed for correlations before logistic regression to build a model of PCa progression. Correlating variables can confound models as it is difficult to ascertain, which variable makes the greater contribution (Kinnear and Gray, 2007). Logistic regression enables the calculation of the natural log (Ln) of the odds of having a more aggressive/indolent disease. From this, the Ln (odds) can be used as a predictor of indolent/aggressive disease. An ROC (receiver operator characteristic) curve was drawn from these values to assess the ability of the model to predict PCa progression and to identify an appropriate cut-off point to distinguish between the two groups.

## Results

### RNA and cDNA quantification and quality

Quantification (absorbance at 260 nm), purity (260/280 and 260/230 nm) and integrity (sighting shot experiment) for each sample are presented in the Supplementary Information (Supplementary Table 3). RNA purity is poorer in those samples with lower quantity, but once reverse transcribed to cDNA, the purity of the samples was improved. Triplicate *PBGD* expression was then used as a measure of cDNA quality. A *PBGD C*_T_ <40 was taken as an indication of adequate quality cDNA. If no amplification was detectable within 40 cycles, it was assumed that there was insufficient starting material.

Following these initial RNA and cDNA analyses, samples were taken forward to be used in the large scale Taqman array study. As a *C*_T_ of <35 is traditionally thought of as optimal, samples that had a *C*_T_ of >35 were loaded onto the Taqman array at 600 ng *μ*l^–1^ rather than at 300 ng *μ*l^–1^ to increase the starting material present. Variation of cDNA starting quantity did not pose a problem as quantification was relative to the amplification of *PBGD* from the same sample.

### Intra- and inter-assay variation

Amplification of cDNA from each sample was performed without replication because of low RNA yield. Therefore, to assess intra- and inter-assay variation, a pooled sample (a mixture of equal volumes of all the samples) was amplified in triplicate at the beginning and end of this research trial. The initial amplification graph of the pooled sample was used to set the threshold fluorescence, above which each amplicon is classed as having ‘come up’ (visible fluorescent signal). The threshold was set at 0.5 so that it was above background noise but still within the exponential phase of amplification (ABI, 2006). A standard curve plate was also included in the trial and consisted of varying concentrations (75, 150, 300 and 600 ng *μ*l^–1^) of the pooled sample and from this the amplification efficiency and *R*^2^ values were calculated.

An optimised qPCR assay should have an efficiency of between 90% and 105% and an *R*^2^ of >0.98 (Bio-Rad, 2006). Of the reference genes included; *18S* was within the correct range for amplification efficiency, *SDHA* and *TBP* were within the correct range for *R*^2^ and *HPRT1* and *PBGD* fell outside those ranges for both values (*R*^2^=0.235 and 0.802, respectively, amplification efficiencies=363.9 and 59.7, respectively). A further measure of qPCR accuracy and reproducibility is the variation between replicates and between different Taqman arrays. Coefficient of variation (CV) was calculated from replicate values within the replicate plates and mean values between replicate plates (Kinnear and Gray, 2007). In order to calculate CVs, *C*_T_ values were converted to actual quantities using the absolute quantification method (see equation in Supplementary Information, Supplementary Table 4). This method uses standard curves of known starting material concentrations to calculate quantity of cDNA. Absolute quantification was used in this calculation to remove the need to normalise to *PBGD* enabling the assessment of *PBGD* reproducibility concurrently. Intra-assay variation was lowest for *SDHA* and *18S*, but inter assay variation was lowest for *PBGD*.

### Quantitative real-time PCR

Initial analysis of qPCR data for reference gene expression showed that only 29 of the initial 41 samples had amplifiable cDNA. Student's *t*-tests were carried out on these 29 samples in both the 2^ΔΔC_T_^ and Pfaffl (see equation in Supplementary Information, Supplementary Table 2) normalised data sets and identified 10 genes that were significantly different between groups divided according to Gleason score (Table 1). Preoperative PSA was also assessed as a predictor of Gleason score. *ANPEP* and *EFNA1* were significantly different in both data sets with 99% confidence, the remaining genes were significant to 95% confidence. All genes showed a significant reduction in expression in aggressive disease compared with indolent disease.

A more accurate method of studying progression of disease is to compare gene expression of patients who have suffered recurrent disease, biochemically or with evidence of distant metastasis, with those who have not. Levels of PSA should be undetectable within 4 weeks of radical prostatectomy (Stephenson *et al*, 2006), so biochemical recurrence was determined by a rising PSA following radical prostatectomy. Evidence of distant metastasis was taken from bone scans. Disease recurrence in all cases was advised by our collaborating clinicians. In this study, between 2 and 5 years clinical follow-up was available for patients. This data analysis identified three genes, *ANPEP*, *INMT* and *TRIP13*; the expression of which was significantly different between patients using both data conversion methods (Table 2). *INMT* and *ANPEP* expression showed a significant decrease (to 99% and 95% confidence, respectively) in recurrent disease while *TRIP13* expression was significantly increased (to 95% confidence) in recurrent disease. Preoperative PSA and Gleason score were also assessed as predictors of recurrence. Gleason score was subjected to a Mann–Whitney *U-*test rather than a Gleason as the data are ordinal.

Genes found to be differentially expressed were then subject to correlation analysis before logistic regression. Correlation analysis was used to identify genes with correlating expression levels as these could not be included in the same logistic regression models. In addition, correlation analysis was performed for gene expression and PSA level before radical prostatectomy and, in the recurrence analysis, Gleason score. Briefly, most genes correlated with each other in the Pfaffl data set, with the exception of *EFNA1, ABL1, GPM6A* and *PSCA*. Further, *INMT* correlated with PSA. The 2^−ΔΔC_T_^ data set showed fewer correlating genes, so most genes could be included in multiple logistic regression analyses. However, *INMT* and *HSPB1* were found to correlate with PSA and *ANPEP* to correlate with Gleason score. Correlation analysis of the genes from the recurrence groupings found that expression levels do correlate across all three genes in both data sets, except *INMT* and *TRIP13* in the 2^−ΔΔC_T_^ data set.

Logistic regression was initially carried out for each gene individually and with PSA and Gleason score (where applicable). The genes with the highest ‘percentage predicted correctly’ were combined to find the best model from the data sets. Table 3 shows the logistic regression data for both data sets divided by Gleason score and recurrence. Analysis of the 2^−ΔΔC_T_^ data set identified *EFNA1* and *ABL1* as good single markers to predict Gleason grouping, a combined model utilising *ANPEP* and *ABL1* as predictors of Gleason grouping and *INMT* as a predictor of recurrence. Analysis of *INMT* and *TRIP13* expression combined as a model of recurrence did not significantly improve the prediction capability over using *INMT* alone. However, combining *TRIP13* with PSA or Gleason score greatly improved the prediction capability of the model. Studying the Pfaffl data set found *HSPB1* to be a good single marker and *ANPEP* and *PSCA* or *CD9* and PSA combined to predict Gleason grouping, whereas *ANPEP* alone or a combined model of *TRIP13*, PSA and Gleason score were identified as the most accurate predictors of recurrence.

**Figure 1:**
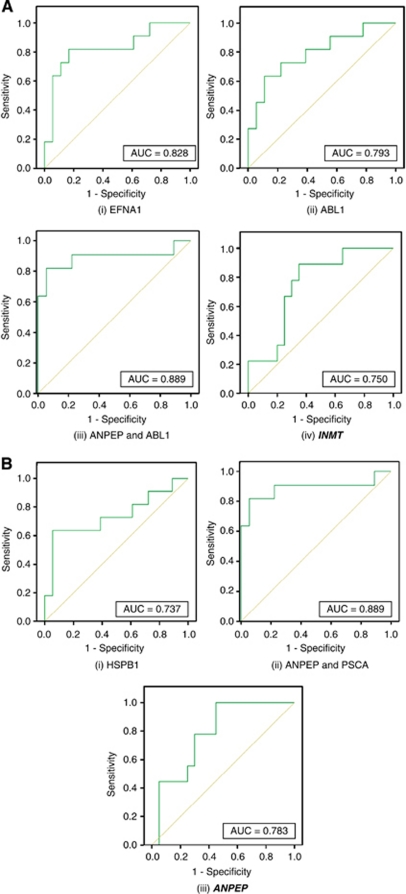
Receiver operator characteristic curves of gene expression as a predictor of PCa progression based on Gleason score and ***recurrence*** using the (**A**) 2^−ΔΔC_T_^ data set and the (**B**) Pfaffl data set. The AUC of each curve is included in the bottom right hand corner of each chart. An ideal AUC should be close to 1.

Receiver operator characteristic curves were drawn for the single and combination markers listed in Table 3. Receiver operator characteristic curves are a further manner of investigating significance and are also used to identify appropriate levels for assigning subjects to one group or another. ROC curves are presented in Figure 1. A good ROC curve is indicated by an ‘area under the curve’ (AUC) of close to one. The coordinates used to produce the ROC curve were used to identify the appropriate gene expression value, which would be used to assign subjects into a particular disease grouping. The cut point is identified as the point at which the sensitivity and specificity are highest and patients with a value below the cut point are more likely to have aggressive disease. The values and the corresponding sensitivity and specificity are detailed in Table 4.

An ideal ROC curve should have high sensitivity and specificity (or low 1-specificity), which means a low probability of false negatives and a low probability of false positives (Kinnear and Gray, 2007). The best ROC curves were produced by the two models using combined expression of two genes, *ANPEP* and *ABL1* (AUC=0.889) in the 2^−ΔΔC_T_^ data set and *ANPEP* and *PSCA* (AUC=0.869) in the Pfaffl data set, for prediction of Gleason score grouping. A combined model using *TRIP13*, PSA and Gleason score in the Pfaffl data set was also very good at predicting recurrence (AUC=0.888).

Within the 2^−ΔΔC_T_^ data set, the best predictive model was a combined gene expression model of *ANPEP* and *ABL1*. This model could correctly assign 89.7% of patients to indolent or aggressive disease groups based on Gleason score. Using a cutoff of −0.16, patients with a higher score could be identified as indolent with 82% sensitivity and 94% specificity. Patients with lower expression of *ANPEP* and *ABL1* could be at risk of potentially aggressive disease. Similarly, the best predictive model within the Pfaffl data set was a combined model of *ANPEP* and *PSCA*, which could correctly assign 86.2% of patients to indolent and aggressive groupings based on Gleason score. A cutoff of –0.25 could predict indolent or aggressive disease groupings with 82% sensitivity and 94% specificity. Again, a score below this cutoff would be indicative of a more aggressive phenotype. Finally, a good predictive model was also found from the Pfaffl data set, a combined model of *TRIP13* with pre-operative PSA and Gleason score of radical was able to correctly assign recurrence in 85.7% of patients. A cutoff of 0.14 could predict recurrence with 87.5% sensitivity and 90% specificity. A score above this cutoff would be indicative of a high chance of recurrence.

Single gene models identified *EFNA1, ABL1* and *HSPB1* as predictors of Gleason score grouping, and *INMT* and *ANPEP* as predictors of recurrence grouping. Both *EFNA1* and *ABL1* (using the 2^−ΔΔC_T_^ data set) had the ability to predict 79.3% of patients as having indolent or aggressive disease. *EFNA1*, with a cut point of −0.40, could predict aggressivity of disease with 82% sensitivity and 83% specificity and *ABL1*, with a cut point of −0.30, could predict aggressivity of disease with 73% sensitivity and 79% specificity. *INMT*, identified using the 2^−ΔΔC_T_^ data set, could predict recurrence of disease in 72.4% of patients and had a sensitivity and specificity of 89% and 65%, respectively, with a cut point of −1.16. Using the Pfaffl data set, *HSPB1* could accurately assign patients into aggressive grouping by Gleason score in 82.8% of cases and using a cut point of −0.54 had a sensitivity and specificity of 64% and 94%, respectively. *ANPEP* could assign patients into recurrence grouping in 79.3% of cases and with a cut point of 0.31 could predict recurrence with 78% sensitivity and 70% specificity. As with the combined gene models, a score lower than the cut point is indicative of aggressive disease.

## Discussion

This study highlights that, because of the complexity of cancer, many genes and proteins are likely to be differentially regulated, and underpins the difficulty of biomarker discovery. Many biomarkers (both diagnostic and prognostic) are identified by researchers but few result in clinically viable tests. Biomarkers that are not significantly better than current testing methods, such as PSA, are unlikely to be embraced by clinicians and, so far, none of the biomarkers discovered have the sensitivity or specificity to replace PSA. Owing to the complexity of PCa (and cancer in general), it is perhaps naive to imagine that a single gene or protein marker will indeed be able to fulfil all of the ‘ideal biomarker’ criteria. This is a ‘proof of concept’ study that highlights the utility of an informed approach when searching for disease biomarkers. Garfield (2000) mentions that sample number is generally limited by resources available for sample collection and sample analysis. For a ‘proof of concept’ study lower sample numbers are generally acceptable, but findings would need to be validated in a larger set of specimens. If validated, the use of these markers could enable clinicians to distinguish between indolent and aggressive forms of the disease and offer informed clinical management options.

Taqman arrays customised for potential PCa progression markers were used to study gene expression in a pilot series of 29 patients. By a variety of data analysis methods, 10 genes were identified, 6 of which could be accurately used to predict PCa progression (defined by Gleason or recurrence) in up to 89.7% of cases. The six genes that were best used to model PCa progression included: *ANPEP, EFNA1, ABL1, INMT, HSPB1* and *PSCA*. *TRIP13* was also an accurate predictor of recurrence when used in conjunction with preoperative PSA and Gleason score.

Aminopeptidase N, or CD13 (ANPEP) is a zinc-dependent matrix metallopeptidase that is membrane bound and thought to have an important role in angiogenesis (Shim *et al*, 2008). Studies of mRNA and protein expression of ANPEP in malignancy are conflicting, with some suggesting an overexpression as an indicator of cancer presence (Razvi *et al*, 2007) and a role for ANPEP in invasion and metastasis (Ishii *et al*, 2001) whereas others propose that reduced expression is associated with cancer (Bogenrieder *et al*, 1997; Wiese *et al*, 2007). A study by Bhagwat *et al* (2001) examined the effect of tumour microenvironment-like conditions on primary endothelial cells and ANPEP production. They found ANPEP to be upregulated in response to hypoxia and increased concentrations of growth factors at both the mRNA and protein level. There are some papers that have studied ANPEP expression in PCa cell lines and suggest that ANPEP shows reduced expression in cancer cells (Dall’Era *et al*, 2007) and that there is a significant reduction in expression between LNCaP and PC3 (which have a more aggressive phenotype) cells (Dozmorov *et al*, 2009). However, there are no studies into ANPEP expression in varying Gleason score PCa tissue so the association of ANPEP with Gleason score is novel. It is tempting to suggest that, as angiogenesis is a feature of early-to-mid stage tumourigenesis, perhaps proangiogenic molecules are downregulated in established poorly differentiated tumours such as Gleason score 7–10 PCa.

The tyrosine kinase, c-Abl, is a protein belonging to the Src family of non-receptor tyrosine kinases that has been implicated in the intrinsic apoptosis pathway, triggered in response to DNA damage (Yamaguchi *et al*, 2010). c-Abl is encoded by the *ABL1* gene whose translocation to the *BCR* gene results in the BCR–Abl fusion protein, known to be involved in the development of some leukaemias (Sirvent *et al*, 2008). In cancers that do not have the *BCR–Abl* translocation, *ABL1* seems to show reduced expression. A study of bladder transitional cell carcinoma found a significant reduction in *ABL1* expression in cancerous compared with normal tissue (Amira *et al*, 2004). In PCa progression, we found *ABL1* to be downregulated. During carcinogenesis, one of the proposed hallmarks is that a cancer cell must evade apoptosis (Hanahan and Weinberg, 2000). By being able to downregulate proapoptotic molecules, such as c-abl, cells can evade apoptosis and continue to proliferate.

Prostate stem cell antigen is a cell surface antigen belonging to the Ly6 family of glycosyl phosphatidylinositol anchored proteins. The Ly6 family consist of at least nine proteins thought to be involved in haematopoietic stem cell development and lymphocyte activation. *PSCA* shows 30% homology to stem cell antigen 2, which has a putative role in prostate development (Moore *et al*, 2008). Generally, PSCA over-expression has been shown to be associated with PCa presence and progression (Han *et al*, 2004; Zhigang and Wenlv, 2004; Lam *et al*, 2005). However, a study by (Schmidt *et al*, 2006) found no relationship between PSCA RNA or protein expression and PCa presence or increasing Gleason score. Another study utilising qPCR to study common PCa markers found a reduction of PSCA RNA expression in malignant compared with benign tissue (Fuessel *et al*, 2003). Further, Moore *et al* (2008) performed a study of tumour development and progression in TRAMP (transgenic adenocarcinoma of the mouse prostate) mice with wild-type *PSCA* and heterozygous and homozygous knockout for *PSCA*. They found at autopsy that a much higher percentage of heterozygous and homozygous *PSCA* knockout mice had metastatic disease. They postulated that PSCA may have a ‘context-dependent function’ with a dual role in PCa progression. Our results support their finding and suggest a role for *PSCA* in aggressive PCa. Perhaps *PSCA* under-expression is associated with the metastatic progression of PCa whereas the over-expression is a feature of the early stages of cellular carcinogenesis.

A variety of developmental processes are implicated in cancer development and progression. Therefore, it is perhaps unsurprising that Ephs and ephrins, which have a role in developmental pathways, are found to be deregulated in carcinogenesis (Fox *et al*, 2006). EFNA1 (ephrin A1) is a ligand that is generally associated with the eph A2 transmembrane receptor and has a putative inhibitory role in angiogenesis and cell growth (Nakamura *et al*, 2005). Over-expression of eph A2 has been shown to be a significant prognostic indicator (Wu *et al*, 2004; Han *et al*, 2005; Nakamura *et al*, 2005). However, Nakamura *et al* (2005) report a negative correlation of eph a2 and EFNA1 expression, with EFNA1 involved in eph a2 degradation and negatively affecting tumour growth (Abraham *et al*, 2006) and VEGF-associated angiogenesis (Ojima *et al*, 2006). Therefore, a decreased level of EFNA1 would be expected to promote cell growth and angiogenesis associated with higher grade PCa. This is in contrast to the findings of ANPEP, a pro-angiogenic molecule. However, both showed decreased expression in this study suggesting that they may be involved in different angiogenic pathways.

Heat shock proteins are expressed ubiquitously in all cells and tissues (Kurahashi *et al*, 2007). HSPB1 (HSP27) is a heat shock protein thought to be involved in cell survival and has been linked to stress-induced apoptosis and invasion in colorectal cell lines (Garrido *et al*, 1997). Our findings of a decrease in *HSPB1* mRNA expression associated with a more aggressive PCa phenotype is in contrast to studies of protein level expression of HSPB1 in PCa (Cornford *et al*, 2000; Kurahashi *et al*, 2007) and breast, ovarian and head and neck cancer (O’Callaghan-Sunol *et al*, 2007). However, a study of oral squamous cell carcinoma found the reverse to be true (Lo Muzio *et al*, 2006). It has been suggested that HSPB1 has a role in the stress response and apoptosis evasion (Huang *et al*, 2007). The results of our study could be merely due to the utilisation of a transcriptomic rather than a proteomic approach suggesting regulation of *HSPB1* may be post-translational rather than at the level of transcription. Studies into the correlation of RNA and protein expression in PCa suggest no significant relationship, or even an inverse relationship, between the two molecular levels (Chen *et al*, 2002).

Indolethylamine *N*-methyltransferase (INMT) is a methylation catalyst involved in the methylation of tryptamine during the production of *N N*-dimethyltryptamine (Jacob and Presti, 2005). This pathway is normally associated with brain activity and psychosis but some studies have found a link between *INMT* expression and cancer. Lian *et al* (2006) studied *PTEN* activity in endometrial cancer and found deregulated expression of *INMT* associated with *PTEN* loss. In another study into non-small cell lung cancer (NSCLC), decreased expression of *INMT* was associated with cancerous tissue when compared with normal lung (Kopantzev *et al*, 2008). Similarly, qPCR data from this study found decreased expression of *INMT* in the more aggressive PCa cases. The implication of the downregulation of *INMT* in association with the progression of PCa is unclear.

TRIP13 is a thyroid receptor interacting protein (Lee *et al*, 1995) whose gene shows copy number changes in 68% of 19 early stage NSCLC tumour samples (Kang *et al*, 2008). *TRIP13* has also been implicated as a marker of early disease related mortality in multiple myeloma as part of a 70-gene model. Further multivariate analysis identified a subset of 17 genes that had a similar predictive power, but *TRIP13* did not feature in this smaller panel (Shaughnessy *et al*, 2007). TRIP13 has been found to interact with CMT2 (Stelzl *et al*, 2005), a key player in the mitosis spindle checkpoint, and is required for cell cycle progression (Habu *et al*, 2002). It is interesting that TRIP13 (by its interaction with CMT2) is implicated in cancer progression as spindle checkpoint defects are known to contribute to loss of chromosome integrity, which can lead to aneuploidy, a common feature of cancer cells (Bharadwaj and Yu, 2004).

The use of multiple biomarkers showed a greater ability to correctly identify aggressive disease than single biomarkers alone and therefore, multivariate analysis may be preferable to univariate analysis. This is unsurprising as cancer is a temporally and spatially complex disease that occurs as a result of several mutations to genes involved in a variety of biochemical pathways. Moreover, carcinogenic mutations may not be identical for each individual. It may be inappropriate to expect a single marker to be able to identify progressive disease with a great deal of accuracy as has been suggested in this study and others (Cheville *et al*, 2008; Kosari *et al*, 2008).

Quantitative real-time PCR has become one of the most widely utilised methods of gene expression analysis in disease onset and progression. Many studies have utilised this technique to validate microarray data. Partheen *et al* (2008) utilised qPCR to validate a panel of seven genes identified via microarray analysis as prognostic factors in ovarian adenocarcinoma. The study yielded four genes as potential biomarkers from a sample of only 19 patients. These data were then verified by looking at protein level expression in a larger cohort. Studies into PCa have also utilised qPCR to study gene expression. Research into a panel of eight putative diagnostic markers identified one gene as a significant predictor of PCa (Rizzi *et al*, 2008), whereas another study began with a panel of nine PCa markers and identified one as diagnostic, but a further three that were indicative of organ-confined disease (Schmidt *et al*, 2006). By using Taqman arrays to study gene expression, it is possible to study larger panels of putative markers than was possible for these studies.

The design of this study may have resulted in potential sample bias as only radical prostatectomy samples were used. Radical prostatectomy tends only to be performed with organ-confined lower grade disease so there is a bias towards low recurrence risk disease. Perhaps a further study of biopsy specimens would overcome any potential bias. An alternative approach might have been to utilise mass spectrometry methods to identify putative protein markers and then design a Taqman array accordingly, but this would be a more biased approach. Additional characterisation could take place at the protein level by immunohistochemical methods and analysis of serum expression via western blotting or ELISA.

In conclusion, this ‘proof of concept’ study presents a putative gene panel of PCa progression. In order to verify any signature, a further study on a large ‘test sample set’ is warranted to investigate disease recurrence or survival and would verify and validate any potential prognostic indicators. Interestingly, these markers were assessed in the mRNA expression data produced by Taylor *et al* (2010) and *ANPEP* and *TRIP13* found to be significantly differentially expressed in terms of Gleason score and biochemical and metastatic recurrence, respectively (Supplementary Data Table 4). *ABL1* was not found to be significant but was approaching significance. Several other genes identified from this study were also verified by Taylor *et al* (2010) as putative markers of PCa progression so should be considered for further study. Taken together, our new data and that within the literature show significant complementarity and will help further our biomarker targeting for the future.

## Figures and Tables

**Table 1:** Genes found to be significantly differentially expressed between Gleason groups <7 and ⩾7 PCa

		**Mean**		
**Gene**	**2**^−**ΔΔC_T_**^ **(*P*-value)**	**Gleason group <7**	**Gleason group** ⩾**7**	**Fold change**
*INMT*	0.015	−0.29	−1.42	4.90
*PSCA*	0.024	−0.75	−2.78	3.71
*ITGB4*	0.021	0.47	−0.90	2.91
*NELL2*	0.018	−0.96	−2.29	2.39
*ANPEP*	0.005	1.36	−1.16	2.17
*EFNA1*	0.006	2.29	1.18	1.94
*ABL1*	0.037	2.05	1.12	1.83
*HSPB1*	0.025	2.18	1.45	1.50
*GPM6A*	0.015	−2.54	−3.54	1.39
*CD9*	0.028	3.47	2.87	1.21
				
	**Pfaffl (*P*-value)**	**Gleason group <7**	**Gleason group ⩾7**	**Fold change**
*PSCA*	0.044	0.01	−2.08	209.00
*ANPEP*	0.011	0.19	−2.30	13.11
*GPM6A*	0.043	1.70	0.27	6.30
*ABL1*	0.028	0.99	−0.30	3.30
*CD9*	0.018	0.69	−0.28	3.46
*INMT*	0.031	0.57	−1.09	2.91
*EFNA1*	0.004	0.55	−0.93	2.69
*HSPB1*	0.037	0.68	−0.47	2.45
*ITGB4*	0.017	0.88	−0.61	2.44
*NELL2*	0.015	0.84	−0.59	2.42
PSA	0.346	6.53	8.06	1.23

Abbreviations: PCa= prostate cancer; PSA= prostate specific antigen.

qPCR data were converted using the 2^−ΔΔC_T_^ and Pfaffl conversion methods and then statistically analysed using Student's *t*-tests. Genes are ordered by descending fold change. Preoperative PSA data have been included for comparison.

**Table 2:** Genes found to be differentially expressed between patients with and without recurrent disease

		**Mean**		
**Gene**	**2^−ΔΔC_T_^ (*P*-value)**	**No recurrence**	**Recurrence**	**Fold change**
*ANPEP*	0.041	0.48	−1.70	4.58
*INMT*	0.010	−0.56	−1.95	3.48
*TRIP13*	0.031	−3.11	−1.70	1.83
				
	**Pfaffl (*P*-value)**	**No recurrence**	**Recurrence**	**Fold change**
*INMT*	0.006	0.14	−1.80	13.64
*ANPEP*	0.03	−0.71	−2.80	3.97
*TRIP13*	0.03	−16.92	7.73	3.19
PSA	0.195	6.81	9.07	1.33
Gleason score	0.051^*^	6.55	7.10	—

Abbreviation: PSA=prostate specific antigen.

^*^P-value calculated by Mann–Whitney *U*-test.

qPCR data were converted using the 2^−ΔΔC_T_^ and Pfaffl conversion methods and then statistically analysed using Student's *t*-tests. Genes are ordered by descending fold change. Preoperative PSA and Gleason score data have been included for comparison.

**Table 3:** Logistic regression analysis of the best progression models

**Data set**	**Groups**	**Single/combined**	**Gene(s)**	**OR (95% CI)**	***P*-value**	**% Predicted correctly**
2^−ΔΔC_T_^	Gleason <7 or ⩾7	Single	EFNA1	4.89 (1.32–18.09)	0.018	79.3
		Single	ABL1	5.49 (1.24–24.27)	0.025	79.3
		Combined	ANPEP	1.80 (1.09–2.98)	0.022	89.7
			ABL1	5.82 (1.117–30.34)	0.036	
	Recurrence	Single	INMT	0.39 (0.15–1.00)	0.050	72.4
		Combined	TRIP13	1.82 (0.94–3.53)	0.078	82.1
			PSA	1.14 (0.90–1.45)	0.269	
		Combined	TRIP13	2.31 (1.02–5.22)	0.044	82.8
			Gleason score	4.97 (1.09–22.77)	0.033	
						
Pfaffl	Gleason <7 or ⩾7	Single	HSPB1	2.11 (1.03–4.29)	0.04	82.8
		Combined	ANPEP	1.69 (1.04–2.74)	0.036	86.2
			PSCA	1.39 (0.93–2.07)	0.111	
		Combined	CD9	2.17 (1.00–4.71)	0.051	82.1
			PSA	0.90 (0.72–1.12)	0.339	
	Recurrence	Single	ANPEP	0.652 (0.43–1.00)	0.048	79.3
		Combined	TRIP13	1.06 (1.00–1.12)	0.036	85.7
			PSA	1.23 (0.90–1.69)	0.200	
			Gleason score	6.31 (1.06–37.52)	0.043	

Abbreviations: CI=confidence interval; OR=odds ratio; PSCA=prostate stem cell antigen; PSA=prostate specific antigen.

Single and combined marker models were assessed using logistic regression for both 2^−ΔΔC_T_^ and Pfaffl data in Gleason and recurrence divided groupings. OR are given (with 95% CIs) of an individual having a higher chance of indolent disease with a lower gene expression level.

**Table 4:** ROC curve cut points as determined by logistic regression analysis

**Data set**	**Groupings**	**Gene(s)**	**Cut point**	**Sensitivity**	**Specificity**
2^−ΔΔC_T_^	Gleason	EFNA1	−0.4028	0.818	0.833
		ABL1	−0.3006	0.727	0.788
		ANPEP + ABL1	−0.1612	0.818	0.944
	Recurrence	INMT	−1.1561	0.889	0.650
		TRIP13 + Gleason score	−0.1524	0.778	0.850
		TRIP13 + PSA	−0.4036	0.625	0.900
					
Pfaffl	Gleason	HSPB1	−0.535	0.636	0.944
		ANPEP + PSCA	−0.2453	0.818	0.944
		CD9 + PSA	0.0015	0.636	0.941
	Recurrence	ANPEP	0.3083	0.778	0.700
		TRIP13 + PSA +Gleason score	0.1390	0.875	0.900

Abbreviations: PCa= prostate cancer; PSCA=prostate stem cell antigen; ROC=receiver operator characteristic.

Sensitivity and specificity values for each ROC curve cut point are also included for each model of PCa progression.
