# Supplementary material for: Identification of markers of prostate cancer progression using candidate gene expression
Source: Br J Cancer. 2011 Nov 10;106(1):157–65. doi: 10.1038/bjc.2011.490 (PMC3251845; doi:10.1038/bjc.2011.490)
Supplement: Supplementary Information [file bjc2011490x1.doc]

**Supplementary information**

**Table 1.** Genes included on the Prostate Cancer TLDA (Taqman low density array). Genes in **bold** are genes identified via ANN (artificial neuronal networks). Where possible, genes have been put into functional groups.

**Table 2.** Equations used during this study. The first equation is the Pfaffl method of data transformation. This was used to transform experimental data for analysis. The second equation is the absolute quantification method used to transfer C_T_ values to absolute values. This was used to assess the reproducibility of the reference genes and study assay variability.

**Table 3.** Nucleic acid quantification and purity data, and mean sighting shot results for PBGD. The ratio of 260/280 gives an indication of nucleic acid quality which should be approximately 1.8 for DNA and 2.0 for RNA. A mean C_T_ of below 40 implies good quality cDNA. N/A means no amplification detected. Sample numbers marked with * are those with a mean PBGD C_T_ of between 35 and 40.

**Table 4.** Data from Taylor *et al* (2010) subjected to Student’s T-test analysis to study the expression of genes from this study in a second data set. BCR = biochemical recurrence and met = metastatic disease. Genes in **bold** are those whose expression change is in agreement with this study.

Table 1

| Target | Alt. names | Accession No. | ABI assay ID |
| --- | --- | --- | --- |
| Oncogene | | | |
| **ERG** | erg-3 | NM_004449 | Hs00171666_m1 |
| **PVT1** |  | M34428 | Hs00413039_m1 |
| **RHBDL1** | RHBDL, RRP | NM_003961 | Hs00610210_g1 |
| **NELL2** | NRP2 | NM_006159 | Hs00196254_m1 |
| EZH2 | ENX-1, EZH1 | NM_004456 | Hs00172783_m1 |
| **SFPQ** | PSF, POMP100 | NM_005066 | Hs00192574_m1 |
| **EEF1A1** | EEF-1, EEF1A, EF-Tu, EF1A, GRAF-1EF, LENG7, PTI1, eEF1A-1 | NM_001402 | Hs00265885_g1 |
| **APOBEC3G** | ARP9, CEM15, | NM_021822 | Hs00222415_m1 |
| TSG | | | |
| **RAP1GA1** | RAP1GAP1, rap1GAPII | NM_002885 | Hs00182299_m1 |
| TGFB2 | TGF-beta2 | NM_003238 | Hs00234244_m1; Hs00236092_m1 |
| SERPINB5 | PI5, Maspin | NM_002639 | Hs00184728_m1 |
| BRCA2 | FACD, FAD, FAD1, FANCB, FANCD, FANCD1 | NM_000059 | Hs00609060_m1 |
| Apoptosis Related | | | |
| **MYC** | c-Myc | NM_002467 | Hs00153408 |
| **ABL1** | ABL, JTK7, c-ABL, p150, v-abl | NM_005157 | Hs00245443_m1 |
| EDNRA | ETA, ETRA | NM_001957 | Hs00609865_m1 |
| HSPB1 | CMT2F, HSP27, HSP28, Hsp25 | NM_001540 | Hs00356629_g1 |
| HSPA5 | BIP, GRP78, MIF2 | NM_005347 | Hs00607129_gH |
| TRA1 | ECGP, GP96, GRP94 | NM_003299 | Hs00427665 |
| Angiogenic | | | |
| **VASH1** | KIAA1036 | NM_014909 | Hs00208609_m1 |
| ANXA2 | ANX2, ANX2L4, CAL1H, LIP2, LPC2, LPC2D, P36, PAP-IV | NM_001002857 | Hs00743063_s1 |
| MMP2 | CLG4, CLG4A, TBE-1 | NM_004530 | Hs00234422_m1 |
| MMP9 | CLG4B, GELB | NM_004994 | Hs003234579_m1 |
| EFNA1* | B61, ECKLG, EFL1, EPLG1, LERK1, TNFAIP4 | NM_004428 | Hs00358886_m1 |
| EFNA2 | ELF-1, EPLG6, HEK7-L, LERK6 | NM_001405 | Hs00154858_m1 |
| EFNA3 | EFL2, EPLG3, Ehk1-L, LERK3 | NM_004952 | Hs00191913_m1 |
| EFNA4 | EFL4, EPLG4, LERK4 | NM_005227 | Hs00193299_m1 |
| EFNA5 | AF1, EFL5, EPLG7, LERK7, RAGS | NM_001962 | Hs00157342_m1 |
| Invasion and Metastasis related | | | |
| **DPT** | TRAMP | NM_001937 | Hs00170030_m1 |
| **GUCY1A3** | GC-SA3, GUC1A3, GUCA3, GUCSA3 | NM_000856 | Hs00168325_m1 |
| **WIF1** | WIF-1 | NM_007191 | Hs00183662_m1 |
| CD9 | BA2, BTCC-1, DRAP-27, GIG2, MIC3, MRP-1, P24, TSPAN29 | NM_001769 | Hs00233521_m1 |
| CDH2 | CDHN, NCAD | NM_001792 | Hs00169953_m1 |
| CDH1 | Arc-1, CDHE, ECAD, LCAM, UVO | NM_004360 | Hs00170423_m1 |
| CD44 | CDW44, ECMR-III, IN, LHR, MC56, MDU2, MDU3, MIC4, MUTCH-I, Pgp1 | AJ251595 | Hs00174139_m1; Hs00153304_m1; Hs00153310_m1 |
| PCDH18 | KIAA1562 | NM_019035 | Hs00251855_m1 |
| CDC42EP3 | BORG2, CEP3, UB1 | NM_006449 | Hs00377831_m1; Hs00272381_s1 |
| CTNNA1 | CAP102 | NM_001903 | Hs00426996 |
| ALCAM | CD166, MEMD | NM_001627 | Hs00233455_m1 |
| ANPEP | CD13, LAP1, PEPN, gp150 | NM_001150 | Hs00174265_m1 |
| ITGB4 |  | NM_000213 | Hs00173995_m1; Hs00174009_m1; Hs00236216_m1 |
| ITGB6 |  | NM_000888 | Hs00168458_m1 |
| ITGA5 | CD49e, FNRA, VLA5A | NM_002205 | Hs00233743_m1; Hs00233732_m1 |
| ITGA3 | CD49C, GAP-B3, MSK18, VCA-2, VL3A, VLA3a | NM_002204 | Hs00233722_m1; Hs00233707_m1 |
| ITGA2 | BR, CD49B, GPIa, VLA-2, VLAA2 | NM_002203 | Hs00158127_m1; Hs00158148_m1 |
| F11R | JAM, JAM-1, JAM-A, JAM1, JAMA, KAT, PAM-1 | NM_144502 | Hs00375889_m1; Hs00170991_m1 |
| TGM2 | TG2, TGC | NM_004613 | Hs00190278_m1 |
| Metabolic | | | |
| **AMACR** | RACE | NM_014324 | Hs00204885_m1 |
| **ALPPL2** | ALPG, ALPPL, GCAP | NM_031313 | Hs00741068_m1 |
| **FADS1** | D5D, FADS6, FADSD5, LLCDL1 TU12 | NM_013402 | Hs00203685_m1 |
| **NP** | PNP, PRO1837 | NM_000270 | Hs00165367_m1 |
| **FABP5** | E-FABP, EFABP, PA-FABP, PAFABP | NM_001444 | Hs02339439_m1 |
| **CYP11A1** | CYP11A, P450SCC | NM_000781 | Hs00167984_m1 |
| **INMT** |  | NM_006774 | Hs00198941_m1 |
| Differentitation and Proliferation | | | |
| GPR12* ? | GPCR21 | NM_005288 | Hs0027037_s1 |
| ASB2 | ASB-2 | NM_016150 | Hs00387867_m1 |
| **C18orf43** | HFL-EDDG1 | NM_006553 | Hs00398895_m1 |
| **HOXC6** | CP25, HHO.C8, HOX3, HOX3C | NM_004503 | Hs00171690_m1 |
| **HPN** | TMPRSS1 | NM_002151 | Hs00170096_m1; Hs01056332_m1 |
| **KCNN4** | IK1, IKCA1, KCA4, KCa3.1, SK4, hIKCa1, hKCa4, hSK4 | NM_002250 | Hs00158470_m1 |
| PSCA | PRO232 | NM_005672 | Hs00194665_m1 |
| **NUP50** | NPAP60, NPAP60L | NM_007172 | Hs00855432_g1 |
| TBRG4 | CPR2, KIAA0948 | NM_030900 | H00229429_m1; Hs00191057_m1 |
| CDKN1C | BWCR, BWS, KIP2, WBS, p57 | NM_000076 | Hs00175938_m1 |
| ITGB1 | CD29, FNRB, GPIIA, MDF2, MSK12, VLAB | NM_002211 | Hs00236976_m1; Hs00559595_m1 |
| **TRAFD1** | FLN29 | NM_006700 | Hs00198630_m1 |
| PTGFR | FP | NM_000959 | Hs00168763_m1 |
| IRF7 | IRF-7H, IRF7A | NM_004031 | Hs00242190_g1; Hs00185375 |
| Detox | | | |
| **GSTM5** | GSTM5-5, GTM5 | NM_000851 | Hs00757076_m1 |
| **GSTM3** | GST5, GSTB, GSTM3-3, GTM3 | NM_000849 | Hs00168307_m1; Hs00356079 |
| Others | | | |
| **C14orf2** | MP68 PLPM | NM_004894 | Hs00191690_m1 |
| **ATP10B** | ATPVB, KIAA0715 | AB018258 | Hs00391638_m1 |
| **RBM12** | SWAN, KIAA0765 | NM_006047 | Hs00246072_s1 |
| **PLCL2** | PLCE2, KIAA1092 | NM_015184 | Hs00392897_m1 |
| POP5 | RPP2, RPP20 | NM_015918 | Hs00210865_m1; Hs00372215_m1 |
| CD4 | CD4mut, T Cell Surface glycoprotein T4 | NM_000616 | Hs00181217_m1 |
| KLHL5 | Kelch-like, Lymphocyte associated activation | NM_015990 | Hs00375006_m1 |
| CD109 | CPAMD7 | NM_133493 | Hs00370347_m1 |
| CTL2 |  | NM_020428 | Hs00220814_m1 |
| CHM | DXS540, GGTA, REP-1, TCD | NM_000390 | Hs00166083_m1 |
| **HLXB9** | HB9, HOXHB9, SCRA1 | NM_005515 | Hs00232128_m1 |
| **TRIP13** | 16E1BP | NM_004237 | Hs00188500_m1 |
| **PRIM2A** | PRIM2, p58 | NM_000947 | Hs00168726_m1; Hs00386277_m1 |
| **MB** | PVALB | NM_005368 | Hs00193520_m1 |
| **NDUFAF1** | CGI-65, CGI65, CIA30 | NM_016013 | Hs00211245_m1 |
| **CRHR1** | CRF-R, CRF1, CRFR1, CRHR, CRHR1f | NM_004382 | Hs00366363_m1 |
| **GPR19** |  | NM_006143 | Hs00272049_s1 |
| **PGC** |  | NM_002630 | Hs00160052_m1 |
| **PLLP** | PMLP, TM4S11 | NM_015993 | Hs00762550_s1 |
| **GPM6A** | GPM6, M6A | NM_005277 | Hs00245530_m1 |
| CANX | CNX, IP90, P90 | NM_001746 | Hs00233492_m1 |
| TERT | EST2, TCS1, TP2, TRT, hEST2 | NM_003219 | Hs00162669_m1 |
| Reference genes | | | |
| HPRT |  | NM_000194 | Hs99999909_m1 |
| PBGD | HMBS | NM_000190 | Hs00609297_m1 |
| SDHA |  | NM_004168 | Hs00417200_m1; Hs00188166_m1 |
| TBP |  | NM_003194 | Hs00427620_m1 |
| ABI reference gene (18S) | | | |

**Table 2**

| Equation | Summary |
| --- | --- |
| Where: E = amplification efficiency | The Pfaffl method for the calculation of the ratio of target gene expression normalised to a reference gene, taking into account amplification efficiencies |
| Where: m = slope of standard curve line  b = intercept of standard curve line | Absolute quantification method for converting C_T_ from qPCR to actual values |

**Table 3**

|  | RNA | |  | cDNA | |  |
| --- | --- | --- | --- | --- | --- | --- |
| Patient ID | Quantity (ng/μL) | 260/280 |  | Quantity (ng/μL) | 260/280 | Mean C_T_ |
| 57 | 3.74 | 1.17 |  | 1706.98 | 1.80 | 34.5 |
| 75 | 15.02 | 2.30 |  | 1655.95 | 1.80 | 31.9 |
| 52* | 5.35 | 1.71 |  | 1739.87 | 1.78 | 35.2 |
| 63 | 11.25 | 1.87 |  | 1540.89 | 1.79 | 33.6 |
| 53 | 13.48 | 1.84 |  | 1741.63 | 1.78 | 34.9 |
| 98 | 25.76 | 1.96 |  | 1700.94 | 1.80 | 30.5 |
| 81 | 29.38 | 1.91 |  | 1751.11 | 1.81 | 31.2 |
| 55 | 9.37 | 1.77 |  | 1682.41 | 1.79 | 31.9 |
| 60* | 9.69 | 1.86 |  | 1666.43 | 1.77 | 36.2 |
| 108* | 30.12 | 2.00 |  | 1711.33 | 1.78 | 35.1 |
| 56 | 10.15 | 2.17 |  | 1855.13 | 1.78 | 34.2 |
| 83 | 2.27 | 1.84 |  | 1755.17 | 1.78 | N/A |
| 70* | 18.52 | 1.99 |  | 1783.62 | 1.78 | 36.0 |
| 105* | 12.25 | 2.03 |  | 1740.37 | 1.78 | 35.7 |
| 92 | 14.30 | 2.03 |  | 1724.74 | 1.78 | 34.8 |
| 78* | 27.90 | 2.10 |  | 1777.28 | 1.78 | 35.6 |
| 100 | 3.81 | 1.78 |  | 2060.22 | 1.80 | N/A |
| 77 | 10.17 | 1.85 |  | 2076.38 | 1.80 | 31.5 |
| 93* | 5.67 | 1.77 |  | 2090.94 | 1.80 | 36.7 |
| 62 | 8.09 | 1.80 |  | 2084.67 | 1.81 | 33.9 |
| 84 | 5.89 | 1.89 |  | 2089.23 | 1.80 | 32.2 |
| 91 | 16.38 | 2.15 |  | 2099.41 | 1.79 | 32.3 |
| 54 | 24.01 | 2.02 |  | 2150.34 | 1.80 | 30.2 |
| 106 | 16.44 | 1.50 |  | 2049.97 | 1.80 | 30.8 |
| 86 | 6.68 | 1.81 |  | 2049.86 | 1.79 | 34.4 |
| 71 | 31.09 | 2.00 |  | 2101.50 | 1.79 | 29.5 |
| 97 | 27.79 | 2.20 |  | 2177.12 | 1.78 | 33.2 |
| 58 | 8.52 | 1.83 |  | 2109.83 | 1.80 | 32.2 |
| 107 | 10.04 | 1.79 |  | 2102.13 | 1.80 | 31.8 |
| 73 | 33.51 | 1.94 |  | 2127.68 | 1.80 | 30.5 |
| 66 | 7.42 | 1.42 |  | 2085.73 | 1.80 | 34.0 |
| 72 | 15.06 | 1.99 |  | 2091.25 | 1.81 | 32.5 |
| 102 | 20.59 | 1.89 |  | 1835.97 | 1.73 | 30.8 |
| 61 | 6.80 | 1.90 |  | 1860.85 | 1.73 | 33.2 |
| 88 | 15.98 | 2.20 |  | 1862.76 | 1.74 | 31.2 |
| 74 | 1.97 | 1.69 |  | 1840.52 | 1.73 | 33.2 |
| 103 | 5.50 | 2.04 |  | 1877.63 | 1.72 | 33.6 |
| 96 | 6.45 | 1.96 |  | 1871.96 | 1.73 | 34.2 |
| 50 | 4.22 | 1.51 |  | 1847.09 | 1.74 | 34.8 |
| 69 | 10.40 | 1.46 |  | 1868.08 | 1.73 | 33.2 |
| 80 | 25.16 | 1.90 |  | 2168.04 | 1.71 | 32.5 |
| 87* | 3.79 | 1.35 |  | 1802.06 | 1.72 | 35.6 |
| 82 | 10.82 | 1.83 |  | 1846.48 | 1.72 | 32.8 |

**Table 4.**

| Gene |  | Mean | |  |
| --- | --- | --- | --- | --- |
|  | p-value | Gleason group <7 | Gleason group ≥7 | Fold Change |
| ***ANPEP*** | 0.002 | 9.97 | 9.03 | 1.10 |
| ***NELL2*** | 0.038 | 6.91 | 6.49 | 1.07 |
| ***GPM6B*** | 0.003 | 7.01 | 6.67 | 1.05 |
| *PSCA* | 0.035 | 8.39 | 8.70 | 1.04 |
| ***CD9*** | 0.024 | 11.23 | 10.98 | 1.02 |
| ***ABL1*** | 0.076 | 9.35 | 9.23 | 1.01 |
| *HSPB1* | 0.754 | 10.87 | 10.84 | 1.00 |
| *ITGB4* | 0.880 | 8.24 | 8.22 | 1.00 |
| *INMT* | 0.865 | 12.76 | 12.76 | 1.00 |
| *EFNA1* | 0.997 | 8.27 | 8.27 | 1.00 |
|  | p-value | No recurrence | BCR and met | Fold Change |
| *ANPEP* | <0.001 | 9.03 | 9.96 | 1.10 |
| ***TRIP13*** | <0.001 | 6.25 | 5.95 | 1.05 |
| *INMT* | 0.906 | 12.76 | 12.76 | 1.00 |
